# Application of genome editing techniques to regulate gene expression in crops

**DOI:** 10.1186/s12870-024-04786-2

**Published:** 2024-02-09

**Authors:** Huirong Dong

**Affiliations:** 1 College of Agronomy and Biotechnology, Yunnan Agriculture University, Kunming, 650201 Yunnan China; 2 Hainan Yazhou Bay Seed Laboratory, Sanya, Hainan, 572024 China

**Keywords:** CRISPR/Cas, Genome editing, Crop improvement, Regulatory regions

## Abstract

**Background:**

Enhanced agricultural production is urgently required to meet the food demands of the increasing global population. Abundant genetic diversity is expected to accelerate crop development. In particular, the development of the CRISPR/Cas genome editing technology has greatly enhanced our ability to improve crop’s genetic diversity through direct artificial gene modification. However, recent studies have shown that most crop improvement efforts using CRISPR/Cas techniques have mainly focused on the coding regions, and there is a relatively lack of studies on the regulatory regions of gene expression.

**Results:**

This review briefly summarizes the development of CRISPR/Cas system in the beginning. Subsequently, the importance of gene regulatory regions in plants is discussed. The review focuses on recent developments and applications of mutations in regulatory regions via CRISPR/Cas techniques in crop breeding.

**Conclusion:**

Finally, an outline of perspectives for future crop breeding using genome editing technologies is provided. This review provides new research insights for crop improvement using genome editing techniques.

## Background

Enormous challenges in crop improvement will be faced in the coming decades to meet the food demands with the global population predicted to reach 10 billion by 2050 [1]. Up to 60% increase in crop production yield is needed to feed this global population [2]. However, global food production suffers from severe challenges due to climate change [3], reduced arable land [4], decreased water resources [5], and biotic and abiotic stresses [6]. Limited source of useful genetic variations for agriculture traits is also a significant obstacle in crop breeding. Therefore, scientific breakthroughs and technological innovations are urgently needed to improve crop production and ensure global food security.

Cross-breeding, mutation breeding and transgenic breeding are the major techniques used for crop improvement for decades [7]. Cross-breeding, which relies on genetic recombination, is labor-intensive and time-consuming [7, 8]. Transgenic breeding technique with genetically modified organisms can be achieved by randomly integrating exogenous DNA into plant genomes [9, 10]. In contrast to transgenic breeding, mutation breeding not only shortens breeding time but also has the advantage of being considered as non-GM [11]. Thus products generated by this approach can obtain governmental agency’s regulatory approval more easily, such as the Clearfield® [12, 13] and Provisia™ series [14] products. Nevertheless, the use of mutagenesis breeding remains limited owing to the random creation of unwanted off-target mutations and the low mutation frequency of on-target genes [15].

Since the elucidation of its biochemical mechanism in 2012 [16, 17], the CRISPR/Cas9 system has been successfully used to accelerate crop improvement and obtain desirable agronomic traits such as increased yield, improved quality, stress tolerance and herbicide resistance, owing to its simplicity, efficiency, and high specificity [18, 19].

This review provides a comprehensive overview of the applications of genome editing technology for crop improvement. The introduction briefly summarizes the development of CRISPR/Cas-mediated genome editing systems and the importance of gene expression regulatory elements. This review will focuses on the development and use of CRISPR/Cas-mediated genome editing techniques in crops through engineering regulatory regions to introduce genetic diversity. Finally, future research directions for the application of CRISPR/Cas-mediated genome editing in crop improvement are discussed.

### Genome editing systems and DNA repair mechanisms

**Fig. 1:**
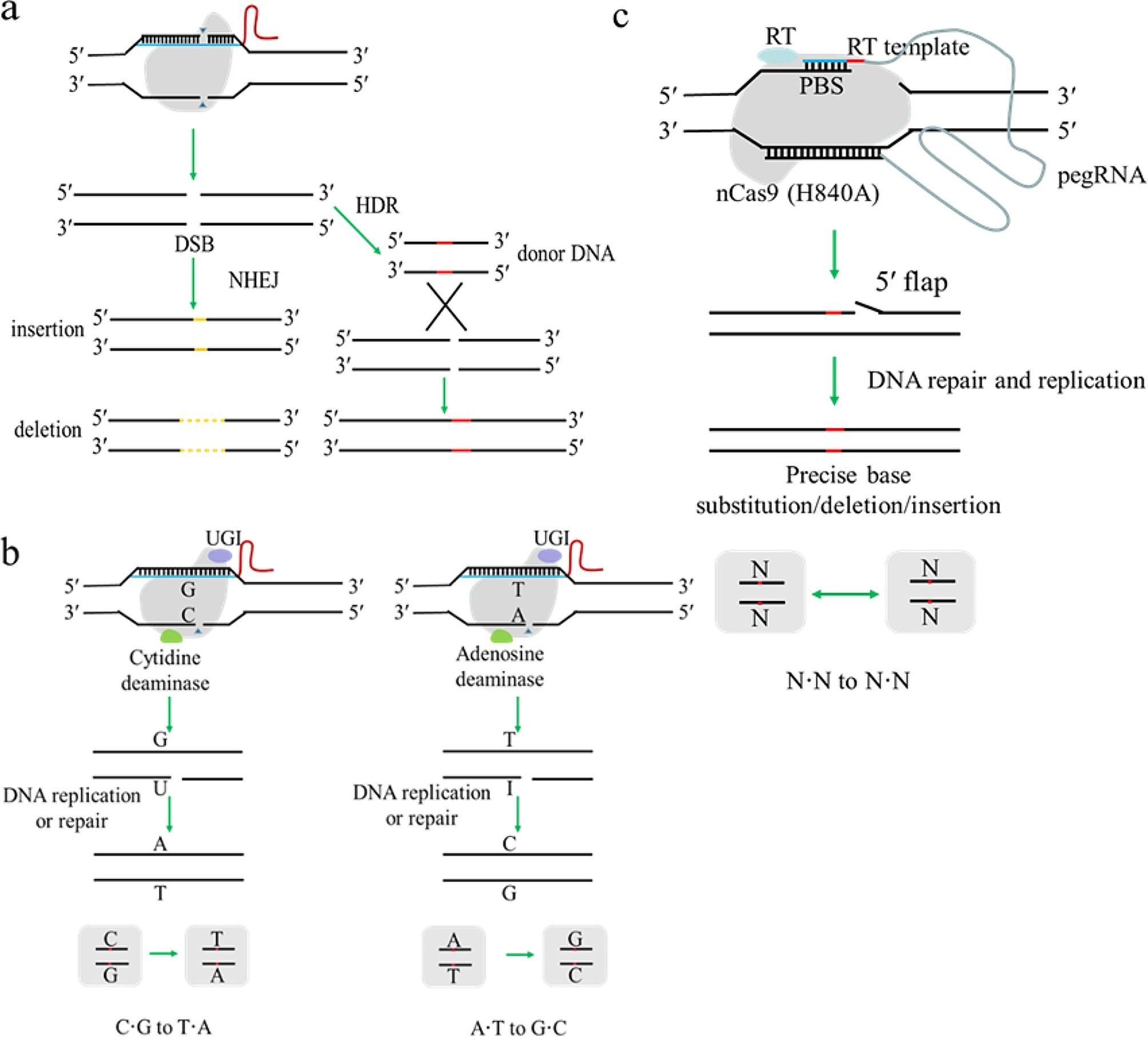
Genome editing systems and DNA repair mechanisms. **(a)** CRISPR/Cas9 system and repair pathways; **(b)** Illustration of CBE and ABE base editors; **(c)** Prime editing system and repair mechanisms

In plants, the CRISPR/Cas-based techniques introduce site-specific double strand DNA breaks (DSBs) that can be repaired via two main pathways: nonhomologous end-joining (NHEJ) and homology-directed repair (HDR) [20] (Fig. 1a). NHEJ is the main DSB repair pathway [21]which depends on an error-prone repair system that often introduces large or small sequence insertions, deletions (indels), or substitutions [22]. HDR-mediated genome editing can achieve precise mutations at a target site, but the efficiency is very low since it is a minor DNA repair pathway in most eukaryotic cells [23].

In addition to NHEJ-mediated and HDR-mediated genome editing, base editors have been developed to induce precise nucleotide substitutions at targeted sites without requiring double strand breaks (DSBs), donor DNA templates, or reliance on homology-dependent repair (HDR) [24, 25]. At present, cytosine base editors (CBEs) and adenine base editors (ABEs), have been developed to catalyze the conversion of C•G base pairs to T•A base pairs [26] and A•T base pairs to G•C base pairs [27], respectively (Fig. 1b). Recently, a novel and precise genome-editing technology, named prime editing, was also developed that enables the introduction of insertions, deletions and all 12 classes of point mutations without requiring DSBs or donor DNA templates (Fig. 1c); prime editor was initially developed in mammalian cells and was shown to function efficiently in plants [28, 29]. To date, these genome editing systems have been successfully used to obtain precise and predictable genome modifications in plants for trait improvement [30].

### Gene expression regulation elements

**Fig. 2:**
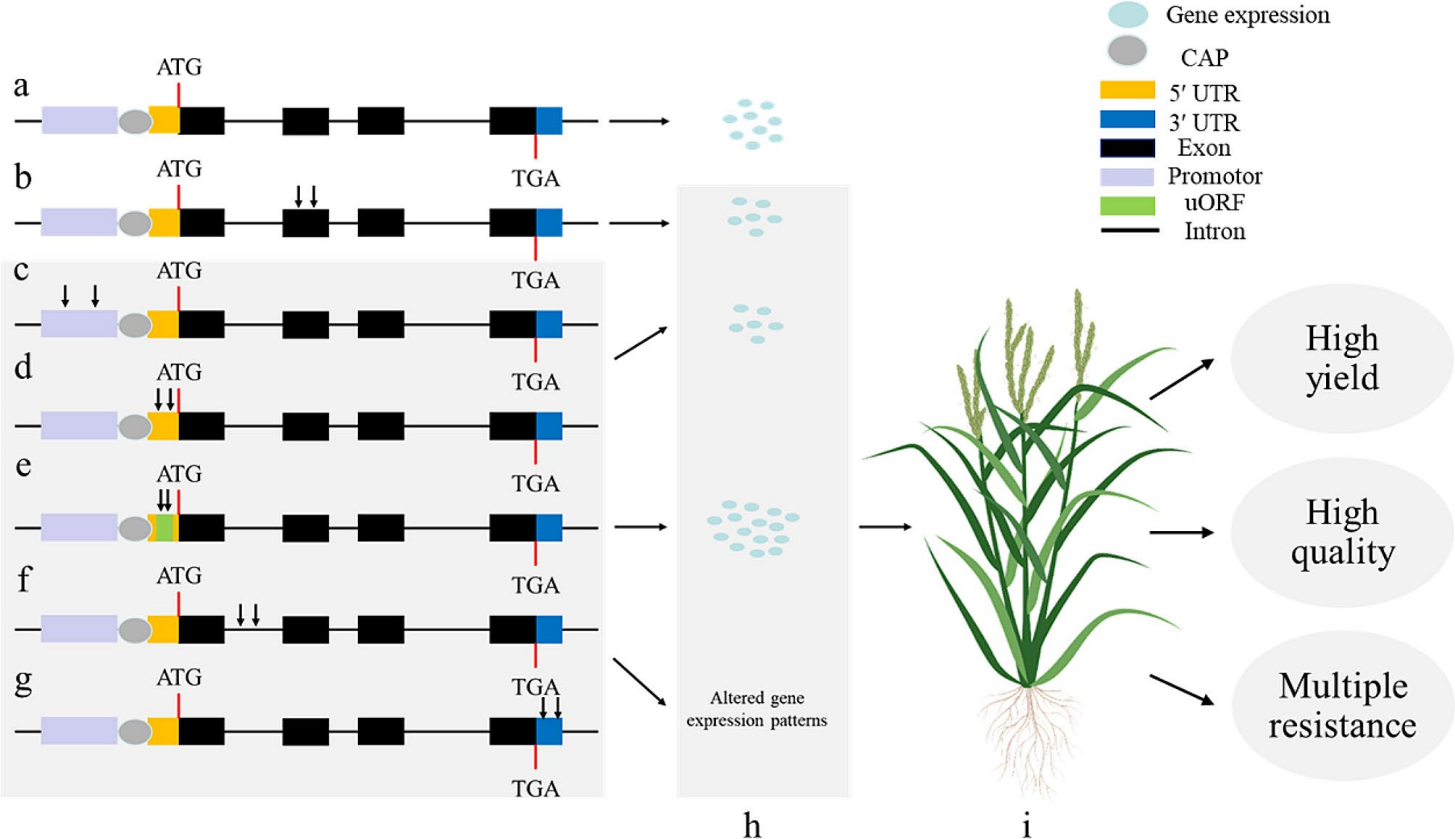
Effect of mutations in the gene coding region and regulatory sequences on gene expression level for crop improvement. **(a)** Expression levels of wild-type plants; **(b)** Coding region mutations have influence on gene expression; **c, d, e, f, g, h.** Mutations in regulatory control regions can affect gene expression levels or expression patterns; **i.** Ideal plants with various elite agronomic traits

Plant growth and development requires precise gene regulation, controlled at the transcriptional, post-transcriptional and translational levels [31]. Up- or down- regulation of gene expression can be achieved by introducing mutations in different regulatory control elements, including promoters, introns, alternative splicing (AS) sites and untranslated regions (UTRs) [32–35]. These approaches for generating novel variations have been applied to crop improvement [36], for example, to accelerate plant domestication and achieve desirable traits by altering gene expression levels without the complete knockout of genes [37] (Fig. 2).

UTRs contain highly conserved structural motifs involved in regulating gene expression. Gene expression and function are regulated by the production of multiple mRNA variants [38–40]. The regulation of gene expression at the translational level is mainly modulated by UTRs [41, 42]. From the cap site to the start codon (excluded) is the 5´UTR that may contain important regulatory elements such as ribosome entry site (RES), secondary structure, upstream ATGs, and upstream open reading frames (uORFs) [43, 44]. These elements may play major roles in translational regulation, including mRNA stabilization, folding, and ribosome interactions. Similarly, the 3´UTR spans from the stop codon (excluded) to the poly-A tail, containing numerous binding sites for regulatory factors [45]. Many regulatory factors involved in the post-transcriptional gene expression regulation regulate biological processes, such as mRNA turnover, localization, and translation efficiency [46, 47]. In addition, 3´UTRs were shown to mediate protein-protein interactions through facilitating alternative protein complex formation, resulting in the diversification of protein functions [48, 49].

Promoters are non-coding DNA sequences that activate gene transcription. Promoters are located at the 5’ upstream of the coding region, containing core promoter regions and upstream promoter response elements that recognize and bind RNA polymerase and transcriptional factors, thereby regulating downstream gene expression [50, 51]. The core promoter region is generally located near the transcription start site, which regulates the transcription initiation of genes, including the transcription start site, TATA-box, initiation factor, and downstream promoter element [52–54]. Promoter response elements, such as enhancers, CAAT-box, GC-box and G-box, are usually located upstream of the core promoter region, and determine the specificity, activity and efficiency of gene transcription [53].

Although introns are not directly involved in the translation of mRNA for protein synthesis, they play an important role in the regulation of gene expression [55]. In many cases, the presence of introns in eukaryotic gene coding regions can improve the gene expression. Introns are excised during post-transcriptional processing [56]. Alternative splicing sites lead to the formation of alternatively spliced mRNA variants, including some with intron retention, which affect gene expression levels or generate alternative protein molecules with different activities [57, 58]. For example, studies have identified a cis-regulatory element CME in the intron of *FLC* gene that can eliminate the “vernalization memory” derived from the parents [59, 60], indicating the importance of introns in the gene expression regulation.

### CRISPR/Cas-mediated editing of regulatory elements for crop improvement

Through numerous studies on development and optimization, CRISPR/Cas systems have demonstrated their power in introducing genetic modification in various crops and potential in accelerating genetic improvement in crops, such as increased yield, improved quality, herbicide resistance, and disease and insect resistance.

### Coding sequence editing for trait improvement

Increasing the crop yield is the primary purpose of crop breeding. The knockout of functional genes, such as *Gn1a*, *DEP1*, *GS3*, and *IPA1*, which negatively regulate yield parameters such as panicle architecture, grain number, grain size, and plant architecture, has been shown to improve rice yield [61–64]. Moreover, better quality requirements rather than higher yields have attracted increasing attention as people’s living standards improve. Mutations in the *Rc* and *SBEII* genes have been used to develop rice lines with increased anthocyanin and amylose contents, respectively [65, 66]. Knocking out the *FAD2* gene in rape increased the oleic acid content in grains [67]. These mutants resulted in greatly improved crop quality.

Furthermore, genome editing techniques also have widespread applications for improving crop resistance traits, especially in herbicide resistance breeding. To date, various crop species that showed tolerance to ALS-inhibiting herbicides have been developed, including rice [68, 69], maize [70], wheat [71, 72], tobacco [73], watermelon [74], oilseed rape [75], tomato, and potato [76]. Similarly, crop resistance to ACCase inhibitors has been developed in wheat and rice [71, 77]. CRISPR/Cas9-directed mutagenesis has also been used to engineer resistance to crop diseases and pests. Tolerance to several catastrophic diseases, such as bacterial blight, blast, and tungro spherical virus, can be generated in rice by disrupting susceptibility genes using CRISPR/Cas9 [78–81]. Mutations in the cytochrome P450 gene *CYP71A1* using the CRISPR/Cas9 technique resulted in resistance to brown planthoppers and borers by suppressing serotonin biosynthesis [82].

However, these mutations occur in the coding region, which may have deleterious pleiotropic effects, and lead to loss-of-function mutants with undesirable plant phenotypes [83]. Thus, engineering gene expression regulatory regions may be a preferred approach for crop improvement (Table 1).

### Disruption of promoters to improve crop traits

Rice production is threatened by several diseases caused by plant pathogens [84]. Bacterial blight, caused by *Xanthomonas oryzae pv. Oryzae (Xoo)*, is a destructive bacterial disease and can reduce rice yield by up to 75%. *SWEET* genes have been reported to be susceptibility genes for bacterial blight [85]. Rice lines with broad-spectrum resistance to bacterial blight were generated by targeting and mutating the promoter region of *OsSWEET* genes [86–88]. Similarly, promoter mutations in the *Xa13* gene, a fully recessive allele related to bacterial blight, using the CRISPR/Cas9 system can also result in bacterial-blight-resistant rice [89]. Moreover, editing the *PthA4* effector binding CREs in the promoter of the susceptibility gene *CsLOB1* in citrus showed enhanced resistance to citrus canker caused by *Xanthomonas citri* subsp. *citri* [90–92].

Amylose content (AC) is a major physiochemical property that determines the eating and cooking quality (ECQ) of rice [93]. AC is governed by the *Waxy* (*Wx*) gene that encodes granule-bound starch synthase I (GBSSI), an enzyme that controls amylose synthesis in the endosperm [94]. Recently, novel *Wx* alleles generated using CRISPR/Cas9-mediated gene knockout resulted in fine-tuned grain AC and produced glutinous rice. Editing promoter or 5´UTR intronic splicing site of the *Wx* gene fine-tuned gene expression at transcriptional and post- transcriptional levels, produced various ACs (amylose contents) by generating diverse quantitative trait alleles in rice and improved the rice quality [95]. Moreover, amylose content can be fine-tuned by creating several novel *Wx* alleles in the japonica cultivar Nipponbare by editing key cis-acting elements that regulate gene expression in the *Wx*^*b*^ promoter using CRISPR/Cas9 technology [96].

Additionally, scientists have identified a wide range of cis-regulatory mutations through targeting the putative *SlWUS CArG* element and *SlCLV3* promoter. A series of mutants were generated with different fruit sizes and quantitative trait variation for elite crop breeding and domestication of wild relatives [97, 98]. Engineering the promoters for quantitative variation of yield-related *CLE* gene using CRISPR-Cas9 genome editing demonstrated the potential of promoter mutations for the modification of grain yield-related trait genes to enhance crop yields [99]. Mutating the key regulatory elements of the main QTL *SLG7* promoter in rice improved *SLG7* gene expression, created new allelic variations with reduced chalkiness, thus providing a new strategy for the rapid improvement of appearance quality of rice [100].

Interestingly, promoter insertion and swapping have great potential for crop improvement via CRISPR/Cas9-mediated HDR. Insertion of the *GOS2* promoter into the 5´UTR or replacement of the promoter of *ARGOS8* gene by CRISPR/ Cas9 technology resulted in increased expression of *ARGOS8* and created drought-resistant maize [101]. Moreover, editing the promoter region may be an effective strategy for reducing the pleiotropic effects. For example, a 54 bp deletion in the promoter of *IPA1*, a gene that showed a trade-off effect between tiller number and panicle size, can simultaneously increase tiller number and panicle size through overcoming their tradeoff, which greatly enhanced grain yield [102]. Mutagenesis in the promoter of *WOX9* gene in tomatoes resulted in multiple pleiotropic phenotypes in both vegetative and inflorescence development, suggesting that targeted promoter mutations using genome editing can reveal conserved gene functions, which can reduce undesirable effects in crop improvement [103].

**Table 1:** Crop traits that have been improved through engineering regulatory regions by genome editing technologies

Regulation region	Crops	Genes	Related traits	Method of editing	References
Promoter	rice	*OsSWEET*	bacterial blight	deletion	[85–88]
	rice	*Xa13*	bacterial blight	deletion	[89]
	rice	*Wx*	amylose content	deletion	[94–96]
	rice	*SLG7*	chalkiness	deletion	[100]
	rice	*IPA1*	grain yield	large deletion	[102]
	maize	*CLE*	grain-yield	deletion	[99]
	maize	*ARGOS8*	drought	replaced	[101]
	tomato	*SlCLV3*	fruit size	deletion	[97, 98]
	tomato	*WOX9*	inflorescence	deletion	[103]
	citrus	*PthA4*	citrus canker	deletion	[90–92]
5’UTR	tomato	*CRTISO*	fruit color	deletion	[104]
uORF	lettuce	*LsGGP*	paraquat resistance	large deletion	[109]
	strawberry	*FvebZIPs1.1*	sugar content	base substitution	[110]
	tomato	*SlGGP1*	ascorbic acid	deletion	[98]
	rice	*OsDLT*	quantitative traits	base substitution	[111]
RNA splicing	*Arabidopsis*	*HAB1*	abscisic acid synthesis	base substitution	[113]
	*Arabidopsis*	*RS31A*	genotoxic	base substitution	[113]
	rice	*Or*	β -carotene	indels	[114]

### Regulating genes expression by mutating UTRs

Untranslated regions (UTRs) are known to regulate gene expression and protein function by producing multiple mRNA variants [40]. Thus, mutations in the UTRs may lead to the development of desirable phenotypes, for instance, deleting the 5´UTR of *CRTISO* gene reduced gene expression, and subsequently causing changes in fruit color in tomato [104].

In plants, about 30% of mRNAs contain uORFs located in the 5´UTR [105], suggesting uORFs are widespread regulatory elements and may play important roles in regulating the downstream mORF (main open reading frame) via translational repression [106, 107]. Therefore, engineering uORFs by genome editing can also be an effective approach for crop improvement [108]. In certain cases, it is easier to upregulate gene expression by altering the uORF than by changing the coding sequences and promoters.

Zhang et al. edited the uORF of several genes, *AtBRI1*, *AtVTC2*, *LsGGP1* and *LsGGP2*, and obtained enhanced mRNA translation of these genes with increased level of the encoded proteins. Moreover, they obtained mutants with paraquat tolerance by designing sgRNAs to modify the two uORFs of *LsGGP1* and *LsGGP2*, respectively [109]. Similarly, editing the highly conserved SC-uORF of the transcription factor gene *FvebZIPs1.1* in strawberries demonstrated a way to increase the sugar content without causing unfavorable agronomic performance [110]. uORF mutations in *SlGGP1* gene that encodes a vitamin C-biosynthetic enzyme substantially increased foliar ascorbic acid levels in wild tomatoes [98]. Recently, Xue et al. generated a suite of uORFs via editing the 5´UTR of *OsDLT* gene involved in the brassinosteroid transduction pathway and obtained various plant heights and tiller numbers in rice [111].

### RNA splicing affects gene expression

Protein expression level is also regulated at the post-transcriptional level. Alternative splicing (AS) of precursor mRNAs (pre-mRNAs) occurs in over 60% of intron-containing genes and plays a critical role in gene expression regulation [112]. The base editing technology is a powerful tool for manipulating plant gene splicing in gene expression regulation and functional studies.

Xue et al. mutated the 5′ splice sites of four genes: *HAB1*, *T30G6.16*, *RS31A* and *Act2* in *Arabidopsis*, and obtained a G-to-A or G-to-C conversion at the desired G of the 5′ splice site and the neighboring G, demonstrating that disruption of the 5’ splice site affected abscisic acid synthesis, toxin response, and intron-mediated enhancement (IME), respectively [113].

Improving the β -carotene content in crops is also an important target of plant breeding. Splice variants in the orange (*Or*) gene play crucial roles in the effective accumulation of β-carotene. Endo et al. changed the splicing junction of *Or* gene at the third exon and intron by genome editing and realized the accumulation of β -carotene in rice callus, and then developed the golden rice lines [114].

## Conclusions and perspectives

During the past decade, CRISPR/Cas-based genome editing technologies coupled with functional genomics have greatly promoted the genetic improvement of crops and have made important progress in improving crop’s yield, quality, and resistance to herbicides, diseases, and pests. However, several challenges remain to be addressed.

At present, unlike mutation in the protein-coding region which often results in the loss of gene function, editing of the gene regulatory regions leads to phenotypic outcomes that are quantitative and may be difficult to assess [115]. In addition, non-homologous end joining (NHEJ), an error-prone repair system that causes random insertions or deletions (indels) near the target sequence, is the predominant repair pathway, which does not support precise modification and often generates unexpected mutations [21, 22]. Although precision genome editors are continuously being developed, especially PE systems that can achieve template-free replacement of bases, the editing efficiency remains low [116, 117]. To overcome these limitations, it is necessary to optimize editing of regulatory region, develop an efficient editing system for precise editing to introduce desirable traits and expand the molecular breeding resources. The newly developed CRISPR-Cas12a promoter editing (CAPE) system introduced quantitative trait variation (QTV) continuums for starch content and grain size by targeting the promoters of *OsGBSS1* and *OsGS3*, respectively, providing an effective strategy for realizing the QTV of important agronomic traits in crops [118].

Moreover, the CRISPR/Cas-mediated mutagenesis of regulatory regions provides a method for inducing subtle phenotypic changes by achieving desirable level of gene expression. Currently, it is difficult to predict the expression levels of edited genes because of many factors, including secondary structure, regulatory elements and upstream and downstream sequences [119]. Therefore, developing a method for predictable endogenous gene expression would be necessary to obtain desired traits for crop improvement. For example, an efficient and easy method has been developed for down-regulating protein translation to predictable and desired levels by engineering uORFs, generating a series of mutants with varied heights and tiller numbers, as predicted, in rice [111]. However, precise prediction tools for other expression regulatory regions (such as promoters, 5′UTR and 3′UTR) need further exploration and development.

Finally, although genome editing technology has been used in various plants, only a few edited products have been successfully commercialized. Though the edited plants may be considered nontransgenic, their social acceptance remains uncertain. The regulatory framework for gene-edited products has not yet been established in many countries. Hence, more efforts are needed to ensure a more favorable environment for regulatory support and public acceptance of genetically edited products.

## Data Availability

Not applicable.
